# Updated 16S rRNA-RFLP method for the identification of all currently characterised *Arcobacter* spp

**DOI:** 10.1186/1471-2180-12-292

**Published:** 2012-12-18

**Authors:** María José Figueras, Arturo Levican, Luis Collado

**Affiliations:** 1 Unitat de Microbiologia, Departament de Ciències Mediques Bàsiques, Facultat de Medicina i Ciències de la Salut. IISPV, Universitat Rovira i Virgili, Reus, Spain; 2 Institute of Biochemistry and Microbiology, Faculty of Sciences, Universidad Austral de Chile, Valdivia, Chile

**Keywords:** *Arcobacter*, Identification, Agarose, Polyacrylamide, 16S rRNA-RFLP, 16S rRNA gene mutations

## Abstract

**Background:**

*Arcobacter* spp. (family *Campylobacteraceae*) are ubiquitous zoonotic bacteria that are being increasingly recognised as a threat to human health. A previously published 16S rRNA-RFLP *Arcobacter* spp. identification method produced specific RFLP patterns for the six species described at that time, using a single endonuclease (*Mse*I). The number of characterised *Arcobacter* species has since risen to 17. The aim of the present study was to update the 16S rRNA-RFLP identification method to include all currently characterised species of *Arcobacter.*

**Results:**

Digestion of the 16S rRNA gene with the endonuclease *Mse*I produced clear, distinctive patterns for 10 of the 17 species, while the remaining species shared a common or very similar RFLP pattern. Subsequent digestion of the 16S rRNA gene from these species with the endonucleases *Mnl*I and/or *Bfa*I generated species-specific RFLP patterns.

**Conclusions:**

16S rRNA-RFLP analysis identified 17 *Arcobacter* spp. using either polyacrylamide or agarose gel electrophoresis. Microheterogeneities within the 16S rRNA gene, which interfered with the RFLP identification, were also documented for the first time in this genus, particularly in strains of *Arcobacter cryaerophilus* isolated from animal faeces and aborted foetuses.

## Background

The genus *Arcobacter,* included in the family *Campylobacteraceae,* has expanded rapidly since it was first recognised in 1991 [1], and currently includes 17 species. Some of these species are considered enteropathogenic to humans and animals, as well as important zoonotic agents. *Arcobacter* species negatively impact the food industry, as many meat products are frequently contaminated with these bacteria, and multiple species have been described from shellfish [2-6]. In addition, the International Commission on Microbiological Specification for Foods classified *A. butzleri* as a serious hazard to human health [7]. However, the true incidence of *Arcobacter* species in environmental and clinical samples is thought to be underestimated because specific detection and identification methods are not normally applied and can be inaccurate [2,8].

A 16S rRNA restriction fragment length polymorphism (RFLP) method for the identification of *Arcobacter* species has previously been described [9]. The method involved a single digestion with the *Mse*I endonuclease and discriminated all *Arcobacter* species that had been described up to 2008, i.e. *A. butzleri, A. cryaerophilus, A. cibarius, A. skirrowii, A. nitrofigilis* and *A. halophilus*[9]. Further molecular methods for the identification of *Arcobacter* species have been reviewed elsewhere [2,9]. Most of the methods described target only the most common species i.e. *A. butzleri*[10,11], *A. cryaerophilus*[12] and/or *A. skirrowii*[13,14]. Even the most recently proposed identification method, m-PCR, described by Douidah *et al*. [15] in 2010, only targeted five species: *A. butzleri, A. cryaerophilus, A. skirrowii, A. cibarius* and *A. thereius*. Furthermore, using this method, the species *A. defluvii*, *A. ellisii*, *A. venerupis* and *A. butzleri* produced an identical and therefore uninformative amplicon [2,5,6].

The limitations of the current methods have arisen because of the limited testing of certain species, as well as the identification of novel species [2,4-6]. Douidah *et al.*[15] suggested that the reliance of the currently-available 16S rRNA-RFLP method on polyacrylamide gel electrophoresis was a major disadvantage for its routine use. Furthermore, the recently described species *A. thereius,* isolated from aborted pig foetuses [16], and *A. trophiarum*, which was recovered from porcine faecal matter [17], produce the same RFLP pattern as *A. butzleri*[2]. Additionally, the new species *A. venerupis*, from clams, produces a pattern that is very similar to *A. marinus*[6,18].

The aim of the present study was to update the 16S rRNA-RFLP identification method to include all the currently characterised species of *Arcobacter*, and to provide protocols for both polyacrylamide and agarose gel electrophoresis so that the method can easily be adapted.

## Results

### *Mse*I digestion can discriminate 10 of the 17 currently described *Arcobacter* species

**Figure 1:**
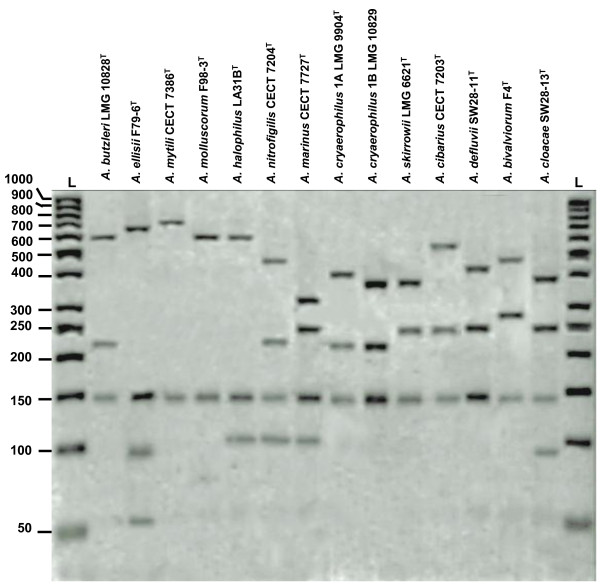
**16S rRNA**-**RFLP patterns (agarose gel 3.5%) obtained for *Arcobacter *spp. using the endonuclease *Mse*I. **Lanes: L, 50 bp ladder, Fermentas. The obtained patterns agree with those expected from the computer simulation (Additional file 1: Table S1). Species that share an identical or similar pattern (Additional file 1: Table S1) were: *A. butzleri*, that produced a pattern identical to those of *A. trophiarum, A. thereius *and atypical strains (n=11) of *A. cryaerophilus*; *A. marinus *CECT 7727^T ^with a pattern very similar to the one of *A. venerupis *CECT 7836^T ^and *A. defluvii* with an identical pattern to the one of *A. suis *strain F41. The identification of these species required additional digestions with other enzymes (Figures 2 – 4, Additional file 2: Table S2 and Additional file 3: Table S3).

Following digestion with the endonuclease *Mse*I, species-specific differential RFLP patterns were obtained for 47 of the 121 strains (38.8%), representing 12 of the 17 species that make up the *Arcobacter* genus (*A. nitrofigilis, A. cryaerophilus, A. skirrowii, A. cibarius, A. halophilus, A. mytili, A. marinus, A. molluscorum, A. ellisii, A. bivalviorum* and *A. venerupis*), including the new described species *A. cloacae* (Figure 1 and Table 1). However, *A. venerupis* produced a pattern very similar to that of *A. marinus*, with only a single 141 bp band distinguishing the two species (Figure 4 and Additional file 1: Table S1). In addition, the new species *A. suis* (F41) showed the same banding pattern as *A. defluvii*, while the characteristic *A. butzleri* pattern (Figure 4 and Additional file 1: Table S1) was also observed following *Mse*I digestion of *A. thereius* and *A. trophiarum* and 11 of the 19 (57.9%) *A. cryaerophilus* strains. Of these, nine strains (MICV1-1, MICV3-2, FE4, FE5, FE6, FE9, FE11, FE13 and FE14) were isolated from animal faeces in Valdivia, Chile, and two strains were isolated in Ireland (LMG 9863 and LMG 9871) from aborted ovine and bovine foetuses, respectively. The RFLP results for these 11 strains were discordant with those of m-PCR and their identity was confirmed by sequencing the 16S rRNA and *rpoB* genes.

**Table 1:** ***Arcobacter *spp**. **strains used in this study**

**SPECIES**	**STRAIN**	**SOURCE**
***A***. ***butzleri***	LMG 10828^T,¶,Ω^, LMG 11118^Ω^	Human faeces
	W24-2-1, W24-05-1, W07-01-8, W03-03-6, W26-02-2, W03-02-7, W21-05-1, W2105-3, W21-05-7, W24-01-1, W10-01-1	Sea water
	SWDS1-3-2	Sewage
	F42, F46^Ω^, F49, F51	Pork meat
	F15, F22, F23, F24, F25	Turkey meat
	F44, F47, F52	Chicken meat
	F43, F50^Ω^, F53	Beef meat
	F1, F2, F29, F30, F38, F98-1, SAN600-1,SAN600-6, SAN512-1, SAN547-10, SAN548-8, SAN582-1, SAN582-6	Mussels
	T62	Soil
***A***. ***trophiarum***	LMG 25534^T,¶,Ω^, LMG 25535^¶,Ω^	Pig faeces
	CECT 7650^Ω^	Chicken cloacae
***A***. ***thereius***	LMG 24486^T,¶,Ω^, LMG 24487^¶,Ω^	Porcine abortion foetus
	SW24^Ω^	Sewage
	F61-1^Ω^	Pork meat
	F89-4	Mussels
	F93-4^Ω^	Clams
***A***. ***cryaerophilus***	LMG 9904^T,¶,Ω^, LMG 9871^¶,Ω^	Bovine abortion foetus
	LMG 9865^¶,Ω^, LMG 10241^¶,Ω^, LMG 6622, LMG 10229^¶,Ω^	Porcine abortion
	LMG 7537^¶^, LMG 9863^¶,Ω^	Ovine abortion foetus
	LMG 10829^¶^	Human blood
	LMG 9861^¶,Ω^	Bovine abortion foetus
	FE4^Ω^, FE5^¶,Ω^, FE6^¶,Ω^, FE9^¶,Ω^, FE11^Ω^, FE13^Ω^	Chicken cloacal swabs
	FE14^Ω^	Ovine faeces
	MICV1-1^¶,Ω^, MICV3-2^¶,Ω^	Cow faeces
***A***. ***nitrofigilis***	CECT 7204^T,¶,Ω^, LMG 7547^Ω^	Roots of *Spartina alterniflora*
	F39^Ω^, F40^¶^, F72^Ω^	Mussels
***A***. ***skirrowii***	LMG 6621^T,¶,Ω^	Lamb faeces
	LMG 9911	Porcine abortion
	Houf 989^¶,Ω^, Houf 994^Ω^	Cow faeces
	S7^Ω^	Sludge
	F94-1^Ω^	Clams
	F125-1^Ω^	Mussels
	ArcoE^Ω^, ArcoF^Ω^	
***A***. ***cibarius***	CECT 7203^T,¶,Ω^	Chicken meat
	NC81^Ω^, NC88^Ω^	Piggery effluent
	H742, H743^Ω^, H745, H746^Ω^, H748	Poultry carcasses
***A****.****halophilus***	LA31B^T,¶,Ω^	Hypersaline lagoon
***A****.****mytili***	CECT 7386^T,¶,Ω^, CECT 7385^¶,Ω^	Mussels
	T234^Ω^	Brackish water
***A****.****marinus***	CECT 7727^T,¶,Ω^	Seawater/starfish
***A****.****defluvii***	CECT 7697^T,¶,Ω^, SW28-7^¶,Ω^, SW28-8, SW28-9, SW28-10, SW30-2^¶,Ω^, SW30-7, SW30-8	Sewage
	MICCC4-2^Ω^	Pig faeces
	SAN599-9^Ω^	Mussels
***A****.****molluscorum***	CECT 7696^T,¶,Ω^, F91^¶,Ω^, F101-1^¶,Ω^	Mussels
***A****.****ellisii***	F79-6^T,¶,Ω^, F79-2^¶,Ω^, F79-7^¶,Ω^	Mussels
***A****.****bivalviorum***	F4^T,¶,Ω^, F118-2^¶,Ω^, F118-4^¶,Ω^	Mussels
***A****.****venerupis***	F67-11^T,¶,Ω^	Clams
***A****.****suis***	F41^T,¶,Ω^	Pork meat
***A****.****cloacae***	SW28-13^T,¶,Ω^	Sewage
	F26^¶,Ω^	Mussels

ATCC American Type Culture Collection, LMG Belgian Co-ordinated Collection of Micro-organisms, CECT Colección Española de Cultivos Tipo.

¶ Sequenced 16S rRNA gene.

^Ω^ Sequenced *rpoB* gene.

### Microhetergeneities in *A. cryaerophilus* strains interfere with RFLP identification

The chromatograms of the 16S rRNA gene sequences (1405 bp) of seven of the 11 unresolved *A. cryaerophilus* strains (MIC V1-1, MICV3-2, FE5, FE6, FE9, LMG 9863 and LMG 9871) showed mutations (i.e. microheterogeneities) at positions 192 (T→C) and 205 (A→G), which were within the target region (TTAA) of the *Mse*I endonuclease (Additional file 4: Figure S1).

### Digestion with *Mnl*I and/or *Bfa*I resolves the remaining species

**Figure 2:**
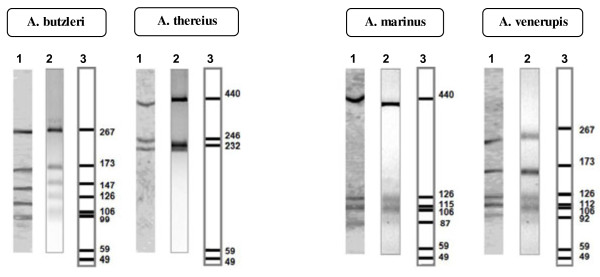
**Species**-**specific 16S rRNA**-**RFLP patterns for species *A.butzleri,A.thereius,A.marinus *and *A****. ****venerupis,* obtained using endonuclease *Mnl*l. **1, polyacrylamide gel 15%; 2, agarose gel 3.5% and 3, computer simulation.

**Figure 3:**
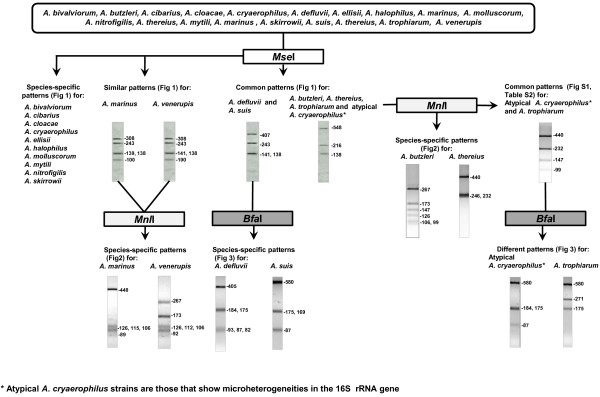
**Species**-**specific 16S rRNA**-**RFLP patterns obtained using endonuclease *Bfa*I for *A.trophiarum,A.cryaerophilus,A.defluvii *and the recently described species *A.suis. ***1, polyacrylamide gel 15%; 2, agarose gel 3.5% and 3, computer simulation.

**Figure 4:**
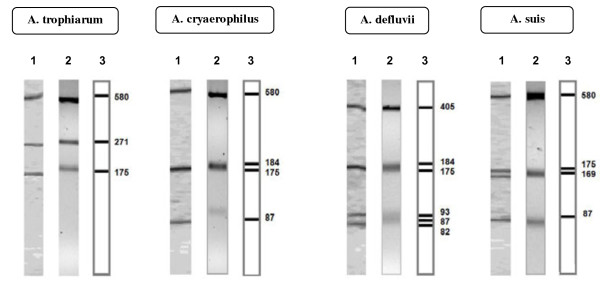
**Flow chart illustrating the proposed order of restriction endonuclease digestions for the 16S rRNA–RFLP analysis for the identification of *Acrobacter *spp.**

A second restriction digest using *Mnl*I (Fermentas) was then carried out for those strains with common or similar RFLP patterns following *Mse*I digestion (Additional file 1: Table S1 and Additional file 2: Table S2). *Mnl*I generated a species-specific pattern for *A. butzleri, A. thereius, A. marinus* and *A. venerupis*, and a common pattern for *A. trophiarum* and the atypical strains of *A. cryaerophilus* (Figures 2 and 4). A further restriction digest step using *FspB*I (Fermentas), an isoschizomer of *Bfa*I, produced species-specific RFLP patterns for the separation of *A. defluvii* from *A. suis* (F41), and *A. trophiarum* from the atypical *A. cryaerophilus* strains (Figure 3 and Additional file 3: Table S3). After carrying out 16S rRNA gene restriction digests as illustrated in Figure 4, all of the 121 strains were correctly identified.

## Discussion

The proposed 16S rRNA-RFLP method described here used an initial digestion with *Mse*I endonuclease, as in the original method [9], which enabled 10 of the 17 accepted species, including the recently described species *A. cloacae*, to be identified. Further digestion was necessary to resolve species that showed the *Mse*I digestion pattern of *A. butzleri* (also common to *A. thereius, A. trophiarum* and to the atypical strains of *A. cryaerophilus* with 16S rRNA gene microheterogeneities). Computer simulation revealed that two endonucleases, *Mnl*I and *Tas*I, produced discriminative patterns between the species *A. butzleri* and *A. thereius* (Figure 2 and Additional file 5: Figure S2). Furthermore, these two enzymes also produced discriminative patterns between *A. marinus* and *A. venerupis* (Figure 2), which showed distinctive but very similar patterns following *Mse*I digestion (Figure 4 and Additional file 1: Table S1). *Mnl*I was selected because it generated more distinctive banding patterns, enabling easier discrimination than *Tas*I (Additional file 5: Figure S2). Considering that *A. butzleri* is a very common species [2,8,19-21], the identification of the majority of strains will normally be obtained with this second (*Mnl*I) endonuclease reaction (Figures 1, 2, 4). In fact, 79.3% of the strains (96/121) included in the current study were correctly identified with this second digestion step.

However, a third digestion, using the enzyme *Bfa*I, was required to distinguish between *A. defluvii* and the recently described species *A. suis* and for distinguishing *A. trophiarum* from the atypical *A. cryaerophilus* strains following *Mnl*I digestion (Figures 3,4 and Additional file 3: Table S3). The proposed method enables reliable and fast species identification for a large collection of isolates, requiring, at most, digestion of the PCR-amplified 16S rRNA gene (1026 bp) with three restriction endonucleases (*Mse*I, *Mnl*I and/or *Bfa*I).

The original 16S rRNA-RFLP method [9] has been used to identify more than 800 *Arcobacter* strains recovered from meat products, shellfish and water in various studies [3-6,19-22]. The existing method has also helped to discover new species on the basis of novel RFLP patterns, including *A. mytili*[3], *A. molluscorum*[4], *A. ellisii*[5], *A*. *bivalviorum, A. venerupis*[6] and *A. cloacae*[23]. Furthermore, as well as identifying the more common *Arcobacter* species, this technique has confirmed the presence of other rare species in atypical habitats, such *A. nitrofigilis* in mussels and *A. thereius* in pork meat [20]. The updated technique described here is likely to supersede the current method in all of these areas.

The use of the 16S rRNA-RFLP method in parallel with the more commonly used molecular identification method, m-PCR [13], as well as the fact that strains with incongruent results were sequenced (*rpoB* and/or 16S rRNA gene sequencing), ensured accurate species identification, and highlighted the limitations of both identification methods [2,4-6,23]. The presence of microheterogeneities in the 16S rRNA gene, as in the case of the 11 atypical *A. cryaerophilus* strains*,* had not previously been observed. These strains produced the m-PCR amplicon expected for *A. cryaerophilus*, which targets the 23S rRNA gene [13], but showed the *A. butzleri* 16S rRNA-RFLP pattern [9]. However, *rpoB* and 16S rRNA gene sequencing results confirmed these strains as *A. cryaerophilus*. 16S rRNA-RFLP patterns that differ from those described here can be expected for any newly discovered *Arcobacter* species [3-6,9,23]. Nevertheless, intra-species nucleotide diversity (i.e. mutations or microheterogeneities in the operon copies of the 16S rRNA gene) at the endonuclease cleavage sites can also generate a novel RFLP pattern for a given isolate, or result in a pattern identical to another species [9,24,25]. In the latter situation, misidentifications may occur, as described here.

## Conclusions

In conclusion, the 16S rRNA-RFLP protocols described here for the identification of *Arcobacter* spp. can be carried out using either agarose or polyacrylamide gel electrophoresis (Figures 1–3, Additional file 1: Table S1, Additional file 2: Table S2, Additional file 3: Table S3), depending on the requirements of an individual laboratory. It is important, however, to carry out the 16S rRNA gene digestions in the order illustrated in the flow chart (Figure 4).

The method provided in this study is reproducible, reliable, simple, fast, and reasonably inexpensive, and can be carried out efficiently in any laboratory. The technique is highly applicable for investigations of the prevalence of arcobacters in a variety of food products, water, wastewater or other environmental samples. It will enable investigators to determine the true incidence of the recently described species *A. mytili, A. marinus, A. trophiarum, A. molluscorum, A. defluvii*, *A. ellisii, A. bivalviorum*, *A. venerupis, A. cloacae* and *A. suis* clarifying their prevalence and epidemiology.

## Methods

### Bacterial strains and culture conditions

A group of 121 *Arcobacter* strains isolated from diverse origins were used in this study, including the type strains of the 17 *Arcobacter* species, as well as strains included in the original descriptions of all species (Table 1). Strains belonging to the most recently described *Arcobacter* species (*A. cloacae*, n=2, and *A. suis*, n=1) [23] were also included in the analysis.

All *Arcobacter* strains were cultured in TSA supplemented with 5% sheep blood at 30°C under aerobic conditions for 48 h in preparation for DNA extraction.

### Strain identification by RFLP

All strains were identified in parallel using the 16S rRNA-RFLP method described by Figueras *et al*. [9] and the m-PCR method of Houf *et al.*[13]. Furthermore, the identities of some strains, especially those that gave either an unknown RFLP pattern, or contradictory results between the two methods (16S rRNA-RFLP and m-PCR), were confirmed by sequencing the 16S rRNA and/or the *rpoB* genes (Table 1) using primers and conditions described previously [3,26].

For the RFLP identification, total genomic DNA was extracted from all strains and used as template for the PCR amplification of a 1026 bp region of the 16S rRNA gene, as previously described [9,27]. 16S rRNA amplicons were digested with *Tru*I (Fermentas, Vilnius, Lithuania), an isoschizomer of *Mse*I, in a 30 μl final volume containing 10 μl of the amplification product, 10 U of the enzyme, 2 μl of 10× buffer, and distilled water. The reaction mixture was incubated at 65°C for 4 h. To separate the restriction fragments, the digested products were electrophoresed on 15% polyacrylamide gels (ProtoGel, Hessle, United Kingdom) at 350 V for 5 h [9], and on 3.5% agarose gels at 100 V for 90 min. In both cases, gels were prepared in 1× Tris-Borate-EDTA (TBE) buffer, and 50 bp ladder (Fermentas) was used as a molecular weight marker. Gels were stained with either SYBR Safe (Invitrogen, Carlsbad, CA, USA) or Red Safe (Ecogen, Barcelona, Spain) DNA gel stains, according to the manufacturers’ instructions, and then photographed on a UV transilluminator Vilber Lourmat Model TFX-35C (Marne-la-Vallée, France).

### Determination of restriction endonuclease recognition sites

Restriction endonuclease recognitions sites within the 16S rRNA sequences of all strains included in this study (Table 1 and Additional file 1: Table S1, Additional file 2: Table S2, Additional file 3: Table S3) were identified using NEBcutter V 2.0 software [28], which is available online (http://tools.neb.com/NEBcutter2/index.php). Experimental validation of the selected enzymes was carried out following the manufacturers’ instructions, under the conditions described above.

## Competing interests

The authors declare that they have no competing interests.

## Authors’ contributions

MJF designed the research project, evaluated results and was principal author. LC isolated the nine strains of *A. cryaerophilus* in Chile and carried out the speciation and 16S rRNA gene mutation analyses. AL carried out the computer simulations, the experimental digestions and participated in the drafting of manuscript under the supervision of LC and MJF. All authors read and approved the final manuscript.

## Supplementary Material

Additional file 1 **Table S1. **Computer simulated profiles of *Arcobacter *spp. 16S rRNA gene (1026 bp) digestion with *Mse*I endonuclease. Species with specific RFLP patterns are in bold. Click here for file

Additional file 4 **Figure S1. **Microheterogeneities (or mutations) in the 16S rRNA gene of seven atypical *A. cryaerophilus *strains in relation to the type strain (LMG 9904^T^), strain LMG 10829 (*A. cryaerophilus* subgroup 1B) and the type strain of*A. butzleri* (LMG 10828^T^). Sequence alignment of the 16S rRNA gene (positions 190–207 in relation to *Escherichia coli*) of seven atypical *A. cryaerophilus *strains showing mutations at positions 192 (T→C) and 205 (A→G), which alter the *Mse*I restriction enzyme recognition site (TTAA). IUPAC code, Y = Pyrimidine (C or T); R = Purine (A or G). Click here for file

Additional file 5 **Figure S2. **Agarose gel (3.5%) comparing the 16S rRNA-RFLP patterns obtained using endonucleases a\) *Tas*I and b) *Mnl*I for species *A. butzleri,A. thereius* and *A. trophiarum*. Lanes 1 and 14, 50 bp ladder (Fermentas); 2, *A. butzleri* LMG 10828^T^; 3, *A. butzleri* F42; 4, *A. butzleri* F43; 5, *A. butzleri* F44; 6, *A. butzleri* F50; 7, *A. butzleri* LMG 11118; 8, *A. thereius* LMG 24486^T^; 9, *A. thereius* SW24; 10, *A. thereius* F89-4; 11, *A.thereius* F93-4 y 12, *A.thereius* LMG 24487; 13, *A. trophiarum* CECT 7650 (identical pattern to that of the 11 atypical strains of *A. cryaerophilus*, Additional file 2: Table S2). *Mnl*I was selected because it produced more distinctive patterns among the species than *Tas*I. Click here for file

Additional file 2 **Table S2. **Computer simulated profiles of *Arcobacter spp.16S rRNA gene * (1026 bp) digestion with *Mnl*I endonuclease. Species in bold are those that show a specific RFLP pattern that was not distinguished with *Mse*I. Click here for file

Additional file 3 **Table S3. **Computer simulated profiles of *Arcobacter *spp. 16S rRNA gene (1026 bp) digestion with *Bfa*I endonuclease. Species in bold are those that now show a specific RFLP pattern that was not distinguished previously with *Mse*I or *Mnl*I. Click here for file
