# Supplementary material for: Updated 16S rRNA-RFLP method for the identification of all currently characterised Arcobacter spp
Source: BMC Microbiol. 2012 Dec 18;12:292. doi: 10.1186/1471-2180-12-292 (PMC3548738; doi:10.1186/1471-2180-12-292)
Supplement: Additional file 1 — Table S1. Computer simulated profiles of Arcobacter spp. 16S rRNA gene (1026 bp) digestion with MseI endonuclease. Species with specific RFLP patterns are in bold. [file 1471-2180-12-292-S1.doc]

**Table S1.** **Computer simulated profiles of the digestion of the 16S rRNA gene (1026 bp) of *Arcobacter* spp. with *Mse*I endonuclease.**

Species with specific RFLP pattern are in bold.

|  | **Presence of 16S rRNA gene RFLP fragments of the following size (bp)^a^** | | | | | | | | | | | | | | | | | | | | | | | | | | |
| --- | --- | --- | --- | --- | --- | --- | --- | --- | --- | --- | --- | --- | --- | --- | --- | --- | --- | --- | --- | --- | --- | --- | --- | --- | --- | --- | --- |
| PesoP | **650** | **615** | **551** | **548** | **519** | **442** | **434** | **407** | **395** | **372** | **366** | **365** | **308** | **269** | **243** | **216** | **167** | **143** | **141** | **139** | **138** | **100** | **99** | **92** | **72** | **52** | **49** |
| *A. butzleri* LMG 10828^T^ |  |  |  | X |  |  |  |  |  |  |  |  |  |  |  | X |  |  |  |  | X |  |  |  |  | X |  |
| *A. thereius* LMG 24486^T^ |  |  |  | X |  |  |  |  |  |  |  |  |  |  |  | X |  |  |  |  | X |  |  |  |  | X |  |
| *A. trophiarum* LMG 24486^T^ |  |  |  | X |  |  |  |  |  |  |  |  |  |  |  | X |  |  |  |  | X |  |  |  |  | X |  |
| *A. cryarophilus* MIC V1-1^b^ |  |  |  | X |  |  |  |  |  |  |  |  |  |  |  | X |  |  |  |  | X |  |  |  |  | X |  |
| ***A. ellisii* CECT 7837^T^** |  | X |  |  |  |  |  |  |  |  |  |  |  |  |  |  |  |  |  |  | X |  |  | X |  | X | X |
| ***A. mytili* CECT 7386^T^** | X |  |  |  |  |  |  |  |  |  |  |  |  |  |  |  |  |  | X |  | X |  |  |  |  | X |  |
| ***A. molluscorum* CECT 7696 ^T^** |  |  | X |  |  |  |  |  |  |  |  |  |  |  |  |  |  |  | X |  | X |  |  |  | X | X |  |
| ***A. halophilus* LA31B^T^** |  |  | X |  |  |  |  |  |  |  |  |  |  |  |  |  |  |  | X |  | X | X |  |  |  | X |  |
| ***A. nitrofigilis* CECT 7204^T^** |  |  |  |  |  |  | X |  |  |  |  |  |  |  |  |  | X |  |  |  | X | X |  |  |  | X | X |
| *A. marinus* CECT 7727^T^ |  |  |  |  |  |  |  |  |  |  |  |  | X |  | X |  |  |  |  | X | X |  | X |  |  | X |  |
| *A. venerupis* CECT 7836^T^ |  |  |  |  |  |  |  |  |  |  |  |  | X |  | X |  |  |  | X |  | X |  | X |  |  | X |  |
| ***A. cryaerophilus* 1A LMG 9904^T^** |  |  |  |  |  |  |  |  | X |  |  |  |  |  |  | X |  | X |  |  | X |  |  |  |  | X |  |
| ***A. cryaerophilus* 1B LMG 10229** |  |  |  |  |  |  |  |  |  |  |  | X |  |  |  | X |  | X |  |  | X |  |  |  |  | X |  |
| ***A. skirrowii* LMG 6621^T^** |  |  |  |  |  |  |  |  |  |  | X |  |  |  | X |  |  | X |  |  | X |  |  |  |  | X |  |
| ***A. cibarius* CECT 7203^T^** |  |  |  |  | X |  |  |  |  |  |  |  |  |  | X |  |  |  |  |  | X |  |  |  |  | X |  |
| *A. defluvii* CECT 7697^T^ |  |  |  |  |  |  |  | X |  |  |  |  |  |  | X |  |  |  | X |  | X |  |  |  |  | X |  |
| *A. suis* F41^T^ |  |  |  |  |  |  |  | X |  |  |  |  |  |  | X |  |  |  | X |  | X |  |  |  |  | X |  |
| ***A. bivalviorum* CECT 7835^T^** |  |  |  |  |  | X |  |  |  |  |  |  |  | X |  |  |  |  |  |  | X |  |  |  |  | X |  |
| ***A. cloacae* SW28-13^T^** |  |  |  |  |  |  |  |  |  | X |  |  |  |  | X |  |  |  |  |  | X |  |  | X |  | X | X |

^a^Small-size bands below 49 bp were not resolved in the electrophoresis and not included in the table.

^b^The same pattern was obtained for 10 other atypical *A. cryarophilus* strains (9 recovered from animal faeces in Chile and 2 from animal abortions in Ireland).
