# Supplementary material for: Updated 16S rRNA-RFLP method for the identification of all currently characterised Arcobacter spp
Source: BMC Microbiol. 2012 Dec 18;12:292. doi: 10.1186/1471-2180-12-292 (PMC3548738; doi:10.1186/1471-2180-12-292)
Supplement: Additional file 4 — Figure S1. Microheterogeneities (or mutations) in the 16S rRNA gene of seven atypical A. cryaerophilus strains in relation to the type strain (LMG 9904T), strain LMG 10829 (A. cryaerophilus subgroup 1B) and the type strain ofA. butzleri (LMG 10828T). Sequence alignment of the 16S rRNA gene (positions 190–207 in relation to Escherichia coli) of seven atypical A. cryaerophilus strains showing mutations at positions 192 (T→C) and 205 (A→G), which alter the MseI restriction enzyme recognition site (TTAA). IUPAC code, Y = Pyrimidine (C or T); R = Purine (A or G). [file 1471-2180-12-292-S4.doc]

**Figure S2**

A._cryaerophilus_LMG_9904T_1A ^190^TGTTAACTTAAGTTAATA^207^

A._cryaerophilus_LMG_10829_1B TATTAACAGAAGTTAATA

A._butzleri_ LMG_10828T TTTTATCAAAAGATAAAA

A._cryaerophilus_MIC_V3-2 TAYTAACTAAAGTTARTA

A._cryaerophilus_LMG_9863 TACTAACCTAAGTTAGTA

A._cryaerophilus_LMG_9871 TACTAACCTAAGTTAGTA

A._cryaerophilus_FE5 TGYTAACTTAAGTTARTA

A._cryaerophilus_FE6 TACTAACATAAGTTAGTA

A._cryaerophilus_FE9 TACTAACAGAAGTTAGTA
