# Supplementary figures and images for: Updated 16S rRNA-RFLP method for the identification of all currently characterised Arcobacter spp
Source: BMC Microbiol. 2012 Dec 18;12:292. doi: 10.1186/1471-2180-12-292 (PMC3548738; doi:10.1186/1471-2180-12-292)

**Figure S1**

**a) 1 2 3 4 5 6 7 8 9 10 11 12 13 14 b) 1 2 3 4 5 6 7 8 9 10 11 12 13 14**


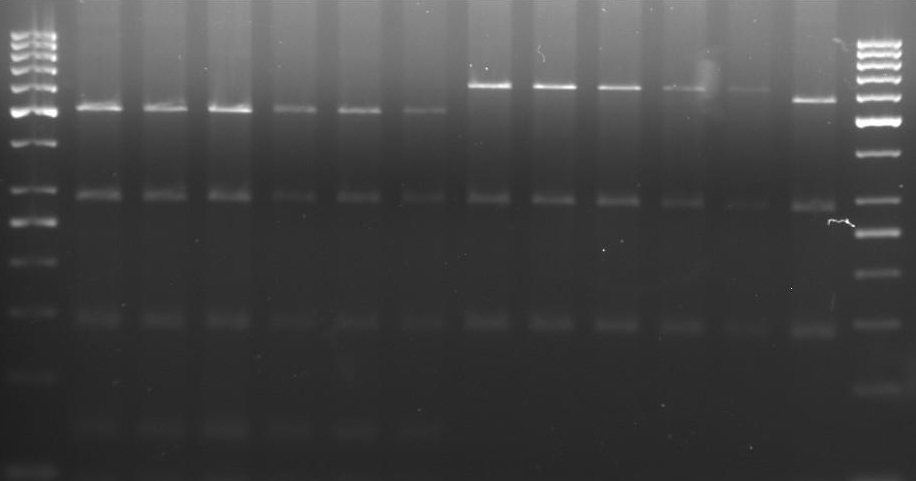

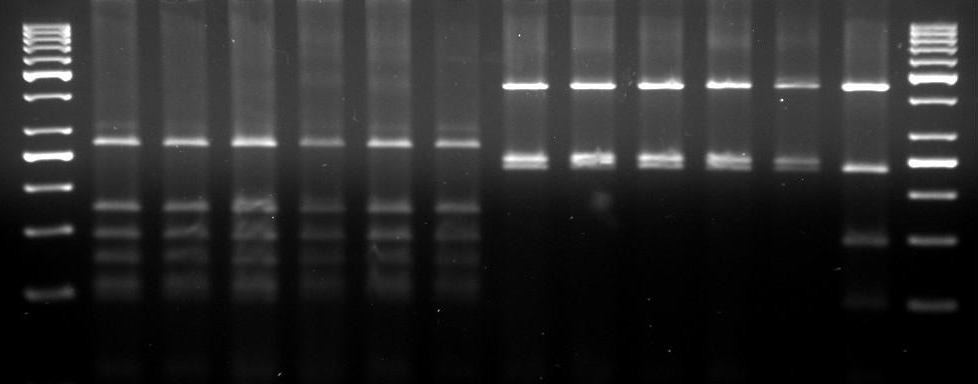

Supplement: Additional file 5 — Figure S2. Agarose gel (3.5%) comparing the 16S rRNA-RFLP patterns obtained using endonucleases a\) TasI and b) MnlI for species A. butzleri,A. thereius and A. trophiarum. Lanes 1 and 14, 50 bp ladder (Fermentas); 2, A. butzleri LMG 10828T; 3, A. butzleri F42; 4, A. butzleri F43; 5, A. butzleri F44; 6, A. butzleri F50; 7, A. butzleri LMG 11118; 8, A. thereius LMG 24486T; 9, A. thereius SW24; 10, A. thereius F89-4; 11, A.thereius F93-4 y 12, A.thereius LMG 24487; 13, A. trophiarum CECT 7650 (identical pattern to that of the 11 atypical strains of A. cryaerophilus, Additional file 2: Table S2). MnlI was selected because it produced more distinctive patterns among the species than TasI. [file 1471-2180-12-292-S5.doc]
