# Supplementary material for: Updated 16S rRNA-RFLP method for the identification of all currently characterised Arcobacter spp
Source: BMC Microbiol. 2012 Dec 18;12:292. doi: 10.1186/1471-2180-12-292 (PMC3548738; doi:10.1186/1471-2180-12-292)
Supplement: Additional file 2 — Table S2. Computer simulated profiles of Arcobacter spp.16S rRNA gene (1026 bp) digestion with MnlI endonuclease. Species in bold are those that show a specific RFLP pattern that was not distinguished with MseI. [file 1471-2180-12-292-S2.doc]

**Table S2.** **Computer simulated profiles of the digestion of the 16S rRNA gene (1026 bp) of *Arcobacter* spp. with *Mnl*I endonuclease.**

Species in bold are those that now show a specific RFLP pattern not distinguished previously with *Mse*I enzyme.

|  |  | **Presence of 16S rRNA gene RFLP fragments of the following size (bp)**^a^ | | | | | | | | | | | | | | |  |  |  |  |
| --- | --- | --- | --- | --- | --- | --- | --- | --- | --- | --- | --- | --- | --- | --- | --- | --- | --- | --- | --- | --- |
|  |  | **440** | **267** | **246** | **232** | **205** | **173** | **164** | **147** | **126** | **115** | **112** | **106** | **99** | **92** | **89** | **87** | **72** | **59** | **49^a^** |
| ***A. butzleri* LMG 10828^T^** |  |  | X |  |  |  | X |  | X | X |  |  | X | X |  |  |  |  | X | X |
| ***A. thereius* L**MG 24486^T^ |  | X |  | X | X |  |  |  |  |  |  |  |  |  |  |  |  |  | X | X |
| *A. trophiarum* L MG 25534^T^ |  | X |  |  | X |  |  |  | X |  |  |  |  | X |  |  |  |  | X | X |
| *A. cryarophilus* MIC V1-1^b^ |  | X |  |  | X |  |  |  | X |  |  |  |  | X |  |  |  |  | X | X |
| *A. cryaerophilus* 1A LMG 9904^T^ |  | X |  |  | X |  |  |  | X |  |  |  |  | X |  |  |  |  | X | X |
| *A. cryaerophilus* 1B LMG 10229 |  | X |  |  | X |  |  |  | X |  |  |  |  | X |  |  |  |  | X | X |
| *A. cibarius* CECT 7203^T^ |  | X |  |  | X |  |  |  | X |  |  |  |  | X |  |  |  |  | X | X |
| *A. skirrowii* LMG 6621^T^ |  | X |  |  | X |  |  |  | X |  |  |  |  | X |  |  |  |  | X | X |
| ***A. marinus* CECT 7727^T^** |  | X |  |  |  |  |  |  |  | X | X |  | X |  |  |  | X |  | X | X |
| ***A. venerupis* CECT 7836^T^** |  |  | X |  |  |  | X |  |  | X |  | X | X |  | X |  |  |  | X | X |
| *A. halophilus* LA31B^T^ |  | X |  |  |  |  |  |  |  | X |  |  | XX | X |  |  |  |  | X | X |
| *A. molluscorum* CECT 7696^T^ |  | X |  |  |  |  |  |  |  | X | X |  | X |  |  | X |  |  | X | X |
| *A. bivalviorum* CECT 7835^T^ |  | X |  |  |  |  |  |  |  | X |  |  | X | X |  |  |  | X |  | X |
| *A. mytili* CECT 7386^T^ |  | X |  | X |  |  |  |  |  | X |  |  | X |  |  |  |  |  | X | X |
| *A. nitrofigilis* CECT 7204^T^ |  |  | X |  |  |  |  | X |  | X | X |  | X |  |  | X |  |  | X | X |
| *A. cloacae* SW28-13^T^ |  |  | X |  |  | X | X |  |  | X |  |  | X |  |  |  |  |  | X | X |
| *A. ellisii* CECT 7837^T^ |  |  | X |  |  | X | X |  |  | X |  |  | X |  |  |  |  |  | X | X |
| *A. defluvii* CECT 7697^T^ |  |  | X |  |  |  | X |  |  | X |  |  | X | X |  |  |  | X | X | X |
| *A. suis*  F41^T^ |  |  | X |  |  |  | X |  |  | X |  |  | X | X |  |  |  | X | X | X |

^a^Small-size bands below 49 bp were not resolved in the electrophoresis and not included in the table.

^b^The same pattern was obtained for 10 other atypical *A. cryarophilus* strains (9 recovered from animal faeces in Chile and 2 from animal abortions in Ireland).
