# Supplementary material for: Updated 16S rRNA-RFLP method for the identification of all currently characterised Arcobacter spp
Source: BMC Microbiol. 2012 Dec 18;12:292. doi: 10.1186/1471-2180-12-292 (PMC3548738; doi:10.1186/1471-2180-12-292)
Supplement: Additional file 3 — Table S3. Computer simulated profiles of Arcobacter spp. 16S rRNA gene (1026 bp) digestion with BfaI endonuclease. Species in bold are those that now show a specific RFLP pattern that was not distinguished previously with MseI or MnlI. [file 1471-2180-12-292-S3.doc]

**Table S3.** **Computer simulated profiles of the digestion of the 16S rRNA gene (1026 bp) of *Arcobacter* spp. with *Bfa*I endonuclease.**

Species in bold are those that now show a specific RFLP pattern not distinguished previously with *Mse*I or *Mnl*I enzymes.

|  |  | **Presence of 16S rRNA gene RFLP fragments of the following size (bp)**^a^ | | | | | | | | | | | |  |
| --- | --- | --- | --- | --- | --- | --- | --- | --- | --- | --- | --- | --- | --- | --- |
|  |  | **580** | **405** | **378** | **271** | **256** | **203** | **184** | **175** | **169** | **93** | **87** | **83** | **62^a^** |
| ***A. trophiarum* LMG 25534**^T^ |  | X |  |  | X |  |  |  | X |  |  |  |  |  |
| ***A. cryaerophilus 1A* LMG 9904^T^** |  | X |  |  |  |  |  | X | X |  |  | X |  |  |
| ***A. cryaerophilus 1B* LMG 10229** |  | X |  |  |  |  |  | X | X |  |  | X |  |  |
| ***A. cryaerophilus* MIC V1-1**^b^ |  | X |  |  |  |  |  | X | X |  |  | X |  |  |
| *A. skirrowii* LMG 6621^T^ |  | X |  |  |  |  |  | X | X |  |  | X |  |  |
| *A. cibarius* CECT 7203^T^ |  | X |  |  |  |  |  | X | X |  |  | X |  |  |
| *A. ellisii* CECT 7837^T^ |  | X |  |  |  |  |  | X | X |  |  | X |  |  |
| *A. cloacae* SW28-13^T^ |  | X |  |  |  |  |  | X | X |  |  | X |  |  |
| ***A. defluvii* CECT 7697^T^** |  |  | X |  |  |  |  | X | X |  | X | X | X |  |
| ***A. suis* F41^T^** |  | X |  |  |  |  |  |  | X | X |  | X |  |  |
| *A. butzleri* LMG 10828^T^ |  | X |  |  | X |  |  |  | X |  |  |  |  |  |
| *A. thereius* LMG 24486^T^ |  | X |  |  | X |  |  |  | X |  |  |  |  |  |
| *A. marinus* CECT 7727^T^ |  | X |  |  | X |  |  |  | X |  |  |  |  |  |
| *A. venerupis* CECT 7836^T^ |  |  |  | X |  |  |  |  | X | X | X | X | X |  |
| *A. halophilus* LA31B^T^ |  |  |  | X | X |  | X |  | X |  |  |  |  |  |
| *A. molluscorum* CECT 7696^T^ |  | X |  |  | X |  |  |  | X |  |  |  |  |  |
| *A. bivalviorum* CECT 7835^T^ |  | X |  |  |  | X |  |  | X |  |  |  |  |  |
| *A. mytili* CECT 7386^T^ |  | X |  |  |  |  |  | X | X |  |  |  |  | X |
| *A. nitrofigilis* CECT 7204^T^ |  |  |  | X |  | X |  |  | X |  | X | X |  |  |

^a^Small-size bands below 62 bp were not resolved in the electrophoresis and not included in the table.

^b^The same pattern was obtained for 10 other atypical *A. cryarophilus* strains (9 recovered from animal faeces in Chile and 2 from animal abortions in Ireland).
